# Recent Advancements in Known and Emerging Risk Factors of Hepatocellular Carcinoma

**DOI:** 10.1002/cam4.71330

**Published:** 2025-11-07

**Authors:** Muhammad Masroor Hussain, Bi Feng, Ju‐Mei Wang, Ao‐Qiang Zhai, Fu‐yu Li, Hai‐jie Hu

**Affiliations:** ^1^ Division of Biliary Tract Surgery, Department of General Surgery, West China Hospital Sichuan University Chengdu China; ^2^ Research Center for Biliary Diseases, West China Hospital Sichuan University Chengdu China

**Keywords:** aflatoxin exposure, gut microbiota and liver cancer, hepatitis B virus (HBV), hepatocellular carcinoma (HCC), immune checkpoint inhibitors, metabolic dysfunction‐associated steatotic liver disease (MASLD), precision medicine in HCC, type 2 diabetes mellitus (T2DM)

## Abstract

**Background:**

Hepatocellular carcinoma (HCC) is the most common primary liver malignancy and a leading cause of cancer‐related deaths worldwide. Despite advancements in antiviral therapies for hepatitis B (HBV) and hepatitis C (HCV), HCC incidence continues to rise due to metabolic dysfunction‐associated steatotic liver disease (MASLD), obesity, type 2 diabetes mellitus (T2DM), and emerging environmental and genetic risk factors. Understanding the evolving landscape of HCC pathogenesis is crucial for improved prevention and treatment strategies.

**Objective:**

This review consolidates recent insights into established and emerging HCC risk factors, highlighting epidemiological trends, molecular mechanisms, and global disparities. It also explores novel therapeutic and preventive strategies aimed at reducing HCC burden and improving patient outcomes.

**Methods:**

A systematic literature review was conducted, incorporating epidemiological studies, molecular research, and clinical trials on HCC risk factors. The interplay between viral hepatitis, metabolic syndrome, environmental toxins, and gut‐liver axis dysregulation was analyzed to provide a comprehensive understanding of HCC development.

**Results:**

While HBV and HCV remain significant drivers of HCC, metabolic risk factors—including MASLD, obesity, insulin resistance, and T2DM—are increasingly prevalent, particularly in Western populations. Environmental exposures such as aflatoxins, alcohol, smoking, and air pollution further exacerbate disease progression. Gut microbiota dysbiosis has also emerged as a key modulator of hepatic carcinogenesis. Advances in precision medicine, including tyrosine kinase inhibitors (sorafenib, lenvatinib), immune checkpoint inhibitors (nivolumab, pembrolizumab), and gut microbiota‐targeted therapies, are transforming HCC management. Early detection is improving through biomarker‐driven surveillance and AI‐enhanced imaging techniques.

**Conclusion:**

The shifting epidemiology of HCC necessitates a multidisciplinary approach to prevention, early detection, and treatment. Integrating genomic profiling, biomarker‐based risk stratification, and equitable healthcare access will be critical to reducing the global burden of HCC.

## 1 Introduction

Hepatocellular carcinoma ranks as the 4th leading cause of cancer all over the world [1]. HCC accounts for the majority of liver cancers, accounting for approximately 90% of all cases [2]. Hepatocellular carcinoma (HCC) typically arises from prolonged liver inflammation, and even though HCV is associated with chronic progression of the disease, HBV can be controlled with new treatments [3]. HCC is the most frequent and a visible leading cause of cancer worldwide [4]. HCC emerges in individuals with a genetic predisposition who encounter risk factors, particularly when liver cirrhosis is present [5]. Many HCC patients are diagnosed at an advanced stage of disease [6]. It's expected that the rates for HCC will rise by 55% by the year 2040 [7]. Each year, over 700,000 new HCC cases are reported globally, with over 600,000 HCC‐related deaths, with a higher incidence in males compared to females [8]. The mortality rate of HCC is increasing worldwide, especially in the Western world. Most cases of HCC occur in China, Southeast Asian countries, and Sub‐Saharan Africa. The highest incidence is in Southeast and Southeast Asia and most often affects people between the ages of 30 and 60 [9]. Cancer is a major health problem in China, ranking second among the six major cancers (blood/lung cancers, liver cancers, stomach cancers, breast cancers, and pancreatic cancer). This study used data directly from the China National Death Surveillance System to survey cancer patients from 2004 to 2018 and found that cancer is the most prominent leading cause of death for the people under the age of 65 yo in China, accounting for 44.35% of all cancers. Cancer ranks first among the leading causes of death. The type of cancer seen in these cases [10]. Cirrhosis still continues to be the most significant risk factor for HCC [11]. The main risk factors of HCC are cirrhosis, HBV, HCV, and long‐term infection or overlap between hepatitis B and hepatitis delta virus (HDV) infection, consumption of aflatoxin‐contaminated foods, continued consumption of alcohol, obesity, smoking, and type 2 diabetes [2, 8, 12]. Viruses such as HBV, HCV, and HDV are important factors for the development of HCC, and non‐communicable diseases are also on the rise. Hepatitis B is considered a major risk factor for HCC and accounts for approximately half (50%) of all HCC cases [13]. Achieving a negative response (SVR) with direct acting antivirals (DAAs) decreases the likelihood of HCV transmission to HCC, but approximately 30% of HCC cases pain is also associated with HCV infection [8, 14, 15]. The presence of both HDV and HBV in a patient with chronic HBV infection will raise the likelihood of HCC by two to six times and speed up the advancement of the disease [8]. Risk factors for HCC vary significantly across various regions. Hepatitis B is a common cause of cancer in various countries in Asia and sub‐Saharan Africa (except Japan); HCV is a common cause of cancer elsewhere [9, 16]. Overall, the Asia Pacific region has the highest rates of hepatitis B and hepatitis C worldwide, with 74% of whole world cancer mortalities occurring in Asia [17]. However, liver fibrosis and cirrhosis in the end turn into cancer. Treatment and management of HCC is difficult because of its poor prognosis and high cancer ratio. The first stage of HCC which is treated with liver resection and other surgical treatments. Chemotherapy, immunot herapy, oncolytic therapy, and nanotechnology can be combined to treat hepatocellular carcinoma and increase effectiveness while reducing side effects. Additionally, combining antibiotics with antibiotics can improve treatment and resistance. Many clinical trials are now showing improved treatment efficacy, reduced relapse rates, and ultimately increased survival [1]. Despite the abundance of published recommendations to help clinicians, there are still areas of controversies and knowledge gaps regarding the diagnosis and treatment of HCC. Ultrasound (US) has a sensitivity of around 63% for detecting HCC, and 6‐month surveillance is better than yearly surveillance [18]. However, the US has limits when it comes to clinical practice, and the presence of obesity, cirrhosis, and/or ascites, as well as the experience and training of technicians, can all have an impact. Less than 20% of cirrhotic patients receive routine monitoring, indicating that surveillance is generally underused [19]. There is debate about the use of biomarkers such des‐gamma‐carboxy prothrombin, AFP‐L3, and alpha‐fetoprotein (AFP). AFP can be coupled with US to optimize early tumor identification; however, this may be linked with greater false‐positive rates and expenses [20]. Another point of contention is whether contrast‐enhanced ultrasonography (CEUS) is a practical and accurate method for diagnosing HCC. The possibility of misdiagnosing it as intrahepatic cholangiocarcinoma (ICC) raised concerns regarding its usage because around 50% of cirrhosis‐related ICCs exhibit the pattern of homogenous arterial hyperenhancement followed by washout on CEUS []. When an imaging‐based diagnosis is ambiguous, a biopsy should be performed, particularly in lesions smaller than 2 cm in diameter, where contrast‐enhanced imaging has inferior diagnostic efficacy. Furthermore, a more active biopsy technique is advised in research settings, as possible consequences of liver biopsy are infrequent and treatable, and refraining from diagnostic biopsy is not justified. The increased availability of liver biopsy in HCC will allow patients to access experimental therapy options while also boosting physicians' understanding of the disease's molecular biology, which will eventually enhance the therapeutic approach to this malignancy in the future. In this review article we tried comprehensively to encapsulate in detail the known and new emerging risk factors, prevention strategies and Risk Assesment/Surveillance Tools for HCC.

## 2 Epidemiology

HCC is the 5th most frequent cancer in men and the 9th most common cancer in women; the male/female ratio is 2.8:1. HCC is formed from hepatocytes, which are responsible for the importance of the heart. HCC accounts for approximately 90% of primary cancer cases worldwide [21]. This disease is commonly diagnosed in people between the ages of 40 and 50, and people with dark skin are diagnosed twice as often as men with light skin [22]. This cancer has a major impact on health and finances worldwide, mainly in Asia. It is considered the 3rd leading cause of cancer in the whole world, having a survival rate of approximately 18% after 5 years [20, 21]. The distribution of HCC varies worldwide, with a higher incidence (more than 20 cases per 100,000 people) occurring in regions where HBV infection occurs, such as Sub saharan africa and East asia. On the other hand, in mediterranean countries i.e., Italy, Greece and Spain, this rate is on average 10–20 people per 100,000 people, while in north and south America this rate is less than 5 people per 100,000 people [22, 23]. Various etiological risk factors may play a very important role in the development of hepatocellular carcinoma. One of the many main causes of liver cancer is the occurrence of liver diseases such as HBV, HCV and HDV, which increases by 2–6 times. Similarly, drinking alcohol and eating foods contaminated with aflatoxin B1 also hike the risk of cancer, which has been connected with an increase in nonalcoholic fatty liver disease (NAFLD) and metabolic disease [26] (Table 1).

**TABLE 1:** Country‐specific HCC incidence and mortality.

Origin	Occurance		Death rate	
	Cases	% ASR	Cases	% ASR
China	410,038	18.2	391,152	17.2
Japan	45,663	10.4	28,155	4.8
Thailand	27,394	22.6	26,704	21.9
Vietnam	26,418	23.0	25,272	21.9
Indonesia	21,392	7.9	20,920	7.7
Korea	14,788	14.3	11,158	9.9
Philippines	10,594	11.4	9953	10.8
North Korea	5607	15.5	5228	14.4
Myanmar	5466	10.0	5281	9.7
Cambodia	3142	24.3	2946	22.9
Mongolia	2236	85.6	2060	80.6
Laos	1272	24.4	1192	22.9
US	42,284	6.9	31,078	4.7

*Note:* Data that GLOBOCAN 2020 has modified.

Abbreviations: ASR, Age‐standardized rate per 100,000; HCC, Hepatocellular Carcinoma.

Although the global age‐standardized occurrence of HCC has been slowly declining since the late 1990s, population growth and aging have led to an increase in the total number of HCC patients. In 2019, 747,000 HCC patients were recorded worldwide, a 70% increase compared to 1990. Age‐related incidence has been increasing since the 1990s in countries with high health indicators, but the incidence has been decreasing worldwide since 2000. The occurrence of liver cancer has increased exponentially in recent years [27].

## 3 Risk Factors

**FIGURE 1:**
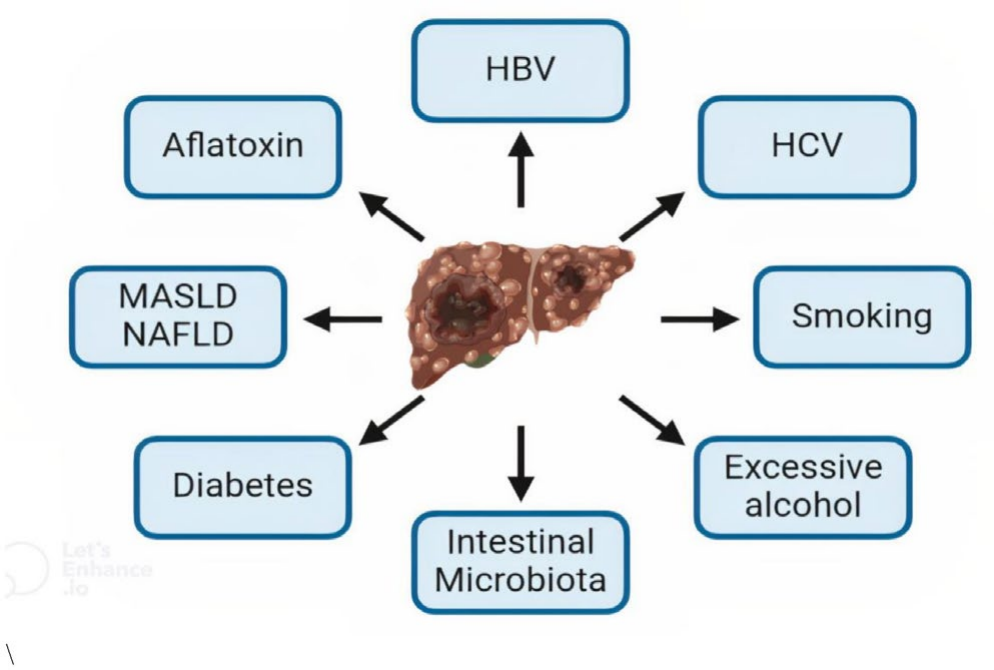
Major HCC risk factors.

The HCC risk factors are mentioned below (Figure 1).

### 3.1 Hepatitis B Virus (HBV)

**FIGURE 2:**
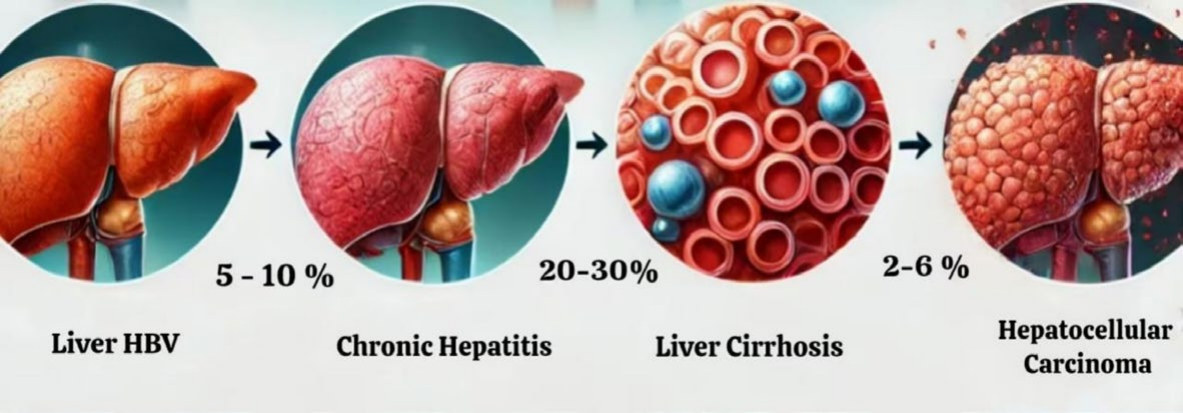
Progress of chronic hbv infection into HCC. This illustration depicting the progression of liver disease, showcasing four distinct stages. The first part shows a healthy liver affected by Hepatitis B Virus (HBV) with slight inflammation. The second part illustrates chronic hepatitis with more noticeable inflammation and fibrosis. The third part shows liver cirrhosis with significant scarring and nodular appearance. Finally, the fourth part depicts hepatocellular carcinoma, highlighting a liver with visible tumors and malignant growths.

HBV infection is a significant worldwide health problem and one of the causes of cirrhosis and HCC [28]. Hepatitis B virus (HBV) infection causes 820,000 deaths each year and is a major burden worldwide [29]. 292 million people are believed to have persistent HBV infection [30]. This sums for more than half of HCC cases in the Asia Pacific region [31]. There is a high burden of disease due to HBV infection in most developed countries, and this remains a global health problem [32]. Only 20% of HCC cases occur in the West, while a great number of HBV cases occur in Asia and Sub‐Saharan Africa where 60% of HBV cases are transmitted [31, 32, 33]. Patients with HBV‐associated cirrhosis have a 44‐fold higher mortality rate and a 31% higher risk for developing HCC [8]. Many other major risk factors that hike the risk for HCC for individuals with HBV include male gender, old age, familial history, type of disease, and cirrhosis. Aflatoxin B1 may also play a significant role in the development of HCC in African HBV‐infected patients by synergizing with HBV to increase the risk of hepatocellular carcinoma (HCC) [2] (Figure 2).

The HBV x protein (HBVx) plays an important part in the growth of HCC because it stimulates Wnt/β‐catenin signaling and encourages the formation of tumors via many antiapoptotic pathways [36]. First, HBVx prolongs the life duration of hepatocytes and induces malignant transformation by inhibiting p53‐mediated apoptosis and upregulating telomerase reverse transcriptase and telomerase activities [37]. In addition, truncation of HBVx facilitates the conversion of HBV into the host genome, and the C terminal region created by this abridgement plays an important role in cancer history. In fact, this chromosomal region promotes hepatocyte growth and oxidative stress by the production of reactive oxygen species (ROS) and deactivates MMP10 through C‐Jun signaling to enhance hepatocellular carcinoma‐associated invasion and metastasis [36, 37, 38]. In West Africa, a case–control research study was carried out to examine the relationship between the severity of liver disease and HBV genetic diversity in a population exposed to the African environment. Regarding virological variables, it demonstrates that HBV genotype A, a minor genotype in West Africa, was substantially linked to the risk of HCC in The Gambia (OR 5.2 [1.3–21.0]). Although carriers of genotype E are less likely to develop HCC than patients with genotype A, this study also discovered that preS2Δ38–55 polymorphisms are linked to a higher risk of HCC in individuals infected with genotype E. This study found the HBV preS2Δ38–55 variation to be a strong independent risk factor and a biomarker for HCC in West African patients, offering new insights into HBV quasi‐species and its effect on the severity of liver disease in The Gambia [41]. With an overall OR of 3.0 (2.3–3.9), a comprehensive literature review and meta‐analysis of 109 similar studies, involving 1511 individuals, has likewise found a link between preS2 mutations and HCC [42].

### 3.2 Hepatitis C Virus (HCV)

**FIGURE 3:**
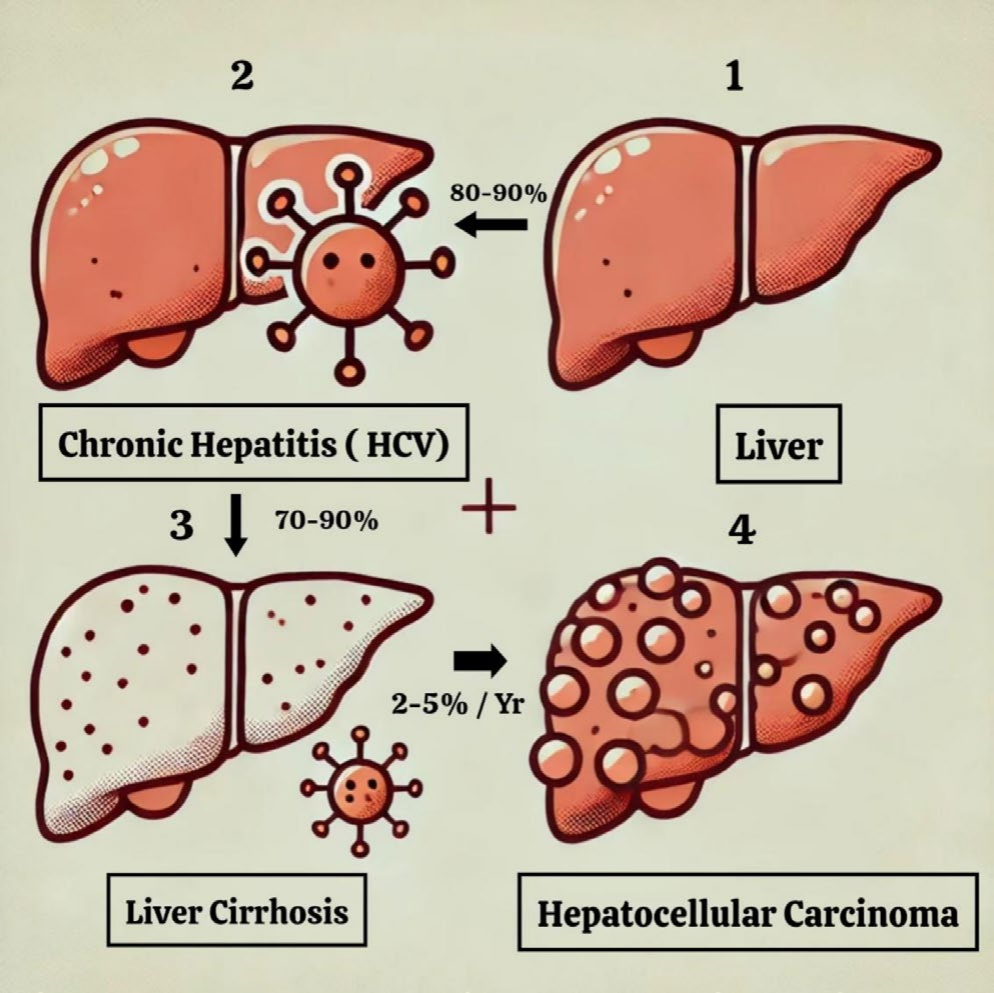
Progress of chronic HCV infection into HCC. An easy‐to‐understand medical illustration of liver disease progression, divided into four sections. The first section depicts HCV virus particles near a healthy liver, the second section shows mild inflammation for chronic hepatitis, the third section displays liver cirrhosis with a nodular texture, and the fourth section illustrates hepatocellular carcinoma with visible tumor growth.

Unlike HBV, HCV does not directly cause genetic changes in the host because it doesn't convert its viral genome into the host genome. The main cause of HCV‐induced HCC is chronic inflammation and weakening of the immune system due to failure to clear the primary infection. These conditions also promote liver fibrosis and cirrhosis, leading to HCC [13]. Approximately 30% of HCC cases are caused by hepatitis HCV, which affects more than 71 million people worldwide [13, 14]. Mostly, HCV infections are chronic because the immune system stimulated by HCV is often weak, weak, and slow, resulting in a weakened immune system [43]. Liver fibrosis is an important stage in the development of HCV‐induced HCC and can lead to the development of cirrhosis. Chronic immune dysfunction and inflammation can eventually promote the formation of liver fibrosis and cause liver damage. Most HCCs often lead to cirrhosis or fibrosis. Even after elimination of HCV, liver fibrosis can progress to HCC, so it needs to be monitored and monitored [44] (Figure 3).

Approximately 1.3–170 million people all over the world have chronic HCV infection. It is estimated that 2.5% of the world's populati on has HCV, and 10%–20% of HCC patients are considered to result from Hcv infection. About 85%–90% of people have chronic HCV infection, and about 5%–10% have HBV infection. HCV is significantly major cause of HCC in developed countries and the major cause of liver transplantation for HCC in the United States. In eastern and African countries the HBV is the biggest risk factor of HCC [45]. HCV is a single stranded RNA virus. Which is a member of the Flaviviridae family that enters human hepatocytes via tight protein s junction (Occludin 1 and Claudin) and/or cell surface receptors like (CD81 and SRB 1) [46]. The HCV genome encodes a polyprotein which is composed of more than 3000 amino acids during the replication process, which subsequently produces different structures (envelope E1, E2 and core) and non structure structures (P7, NS‐2, NS‐3, NS‐4A, NS‐4B, NS‐5A and NS‐5B) [47]. When HCV induced chronic inflammation occurs in infected hepatocytes, proinflammatory and fibrotic mediators including reactive oxygen species and cell mortality signals are released [48]. Liver inflammation and cirrhosis are promoted by intracellular inflammatory initializing and activation of hepatic stellate cells caused by nuclear receptor family (peroxisome proliferator‐activated receptor, farnesin alcoholX receptor, etc.) treatment. However, this optimization also promotes the turnover of liver cells [49]. Many studies have shown that essential proteins can modulate the MA Pk pathway and thus promote cell division. In addition, NS5A inhibits p53 protein and causes tumor degeneration, which is unfavorable for its antitumor effect [50]. In addition, the strongest mitogenic signal is platelet‐derived growth factor (PDGF), which promotes fibrogenesis by expressing β PDGF re ceptors in stellate cells as well as other growth factors on the cell surface including integrins. In turn, HCV replication induces the host immune response, leading to fibrosis and end‐stage liver disease via tumor necrosis factorα and interferon. The severity of fibrosis is straightly related to the risk of developing hcc, but many studies show that HCC can be detected even with low or no fibrosis [51]. In patients with chronic infection with hepatitis C virus (HCV), successful treatment with direct acting antivirals (DAA) reduces HCC risk. A research was carried out in the United States, using data gathered from the Central Cancer Registry (CCR) and the Corporate Data Warehouse (CDW) of the US Department of Veterans Affairs (VA). Patients who attained SVR with DAA at any of the 130 VA hospitals and were at least 18 years old were included. During the mean 2.51 (SD = 1.66) years of follow‐up (maximum 7.11 years), 3247 incident cases of HCC were reported; 74.0% (*n* = 2404) of these were detected in patients who had cirrhosis at baseline. At 1, 2, and 3 years, the cumulative incidence of HCC in 29,398 cirrhosis patients was 2.0%, 3.8%, and 5.4%, respectively. On the other hand, 843 of the 63,169 individuals without cirrhosis who received an HCC diagnosis had a much decreased probability of developing the disease (0.2%, 0.5%, and 0.8%, respectively) (*p*‐value < 0.0001) [51].

### 3.3 Alcohol

Around the world, alcohol‐related liver disease is the most common form of chronic liver disease, contributing approximately 30% of cases of hepatocellular carcinoma (HCC) and deaths from HCC alone. The biggest risk factor for HCC is alcohol consumption, especially alcohol consumption (50–70 g per day) [52]. It's also thought to be the leading cause of death among people aging 15–49 years in developed Western countries [25]. Alcohol dehydrogenase and CYP2E1 metabolize alcohol to acetaldehyde at low and high alcohol concentrations, respectively. Aldehyde dehydrogenase then metabolizes the alcohol to acetic acid. Damage to hepatocytes is caused by ethanol metabolites, especially large amounts of reactive oxygen species (ROS), causing oxidation of proteins, lipids, and DNA. In addition, direct cellular damage is caused by lipid peroxidation, DNA damage, and signals sent by nuclear factor kappa B of the tumor necrosis factor (TNF) pathway. There is no evidence that drinking alcohol directly causes cancer. On the other hand, some meta‐analyses have shown that alcohol is connected with hepatitis B virus and hepatitis C virus disease and the development of cirrhosis and fibrosis, as well as with people drinking more. Considering all regions of the World Health Organization, alcohol consumption per adult has increased by approximately 10% in the last 25 years. This growth was mainly driven by significant growth in Asia (especially China and India) and Africa, while consumption in North America, South America, and Europe declined by 1% and 10%, respectively [53]. Alcohol was thought to be a factor in 19% of liver cancer cases worldwide in 2019; this rate is lower than the percentage of deaths due to HBV (40%) and HCV (29%) [54]. Alcohol is the second largest factor in age‐standardized deaths from liver cancer worldwide, largest in the US. Per capita global alcohol consumption is expected to increase due to economic expansion, especially in highly populated countries in the Western Pacific and Southeast Asian regions [55]. According to a recent meta‐analysis, the cumulative incidence of HCC in patients with ALD‐cirrhosis was 1% at 1 year, 3% at 5 years, and 9% at 10 years, which is less than the 1.5% yearly incidence threshold at which monitoring is deemed cost‐effective. ALD‐cirrhosis had a lower risk of developing HCC compared to MASLD‐cirrhosis (8 studies, 20,602 patients; relative risk 0.65; 95% CI 0.44–0.95; *p* = 0.03) and HCV‐cirrhosis (6 studies, 1056 patients; relative risk 0.29; 95% CI 0.23–0.37; *p* < 0.001). However, the incidence rate per 1000 person‐years was considerably higher in studies involving patients undergoing HCC surveillance compared to those not receiving surveillance, showing that HCC surveillance may be cost‐effective for patients enrolled in dedicated surveillance programs [56]. Therefore, better strategies should be adopted to reduce alcohol consumption. Several investigations utilizing Danish health registries have revealed that people with ALD had significant rates of competitive death [57]. In ALD, genetic propensity to HCC is considered to entail polymorphisms impacting practically all ethanol metabolism routes. Some single nucleotide polymorphisms, including rs738409 (PNPLA3), rs58542926 (TM6SF2), rs187429064 (TM6SF2), rs72613567 (HSD17B13), rs429358 (APOE), and rs641738 (MBOAT7), have emerged as important susceptibility loci for ALD‐HCC [56].

### 3.4 Aflatoxin

Aflatoxin is known as Aflatoxin and Aflatoxin's secondary metabolite. Found mostly in Asia and Sub‐Saharan Africa, these fungi thrive in moist, warm soil. They also contaminate foods, especially those found in wheat, rice, soybeans, corn, and peanuts. Kojitoxin B1 (AFB1) cancer research [56, 57]. In fact, although AFB1 is taken up by enterocytes of the small intestine, injury to the liver is associated with the conversion of aflatoxin 8, 9 exoepoxide by hepatic cytochrome‐P450. Animal studies originally showed that AFB1 was hepatotoxic and fatal at high dosages. Human research stalled owing to a lack of data on aflatoxin metabolism and susceptibility factors. Dietary evaluations were insufficient for establishing an AFB1‐HCC relationship due to the variability of food contamination. However, the introduction of aflatoxin‐specific biomarkers allowed research to provide conclusive proof of this connection [60]. Two cohort studies in Shanghai, China, used AFB1 biomarkers to investigate urine levels of AFB1 and serum levels of HBV among men. The study discovered that men who tested positive for AFB1 had a 3.4‐fold greater risk of liver cancer, a 7‐fold higher risk of HBV, and a 59‐fold higher risk of combined AFB1 and HBV [61]. Several years later, a follow‐up cohort research from Taiwan verified the results [62]. According to studies conducted in China and Taiwan, AFB1 is particularly carcinogenic when co‐occurring with chronic HBV infection, as the two factors have a synergistic effect on HCC risk [59, 60]. According to a 2012 meta‐analysis, AFB1 raised HCC risk by 6‐fold, HBV by 11‐fold, and the two variables together by 54‐fold [63].

More than 50% of HCC is associated with aflatoxin exposure. This metabolite is more active with the p53 tumor suppressor gene, causing a codon 249 mutation nucleotide change [62, 63].

### 3.5 Metabolic Factors

#### 3.5.1 MASLD/NAFLD

NAFLD is the most prevalent disease in already developed countries, having an incidence of 6%–35%. In addition, it is considered one of the significant factors in the incidence of CLD, with rates 20%–30% higher than in Western countries (5%). With the significant progress in recent years, including changes in terminology, FLD is associated with metabolic dysfunction (MASLD). It is the newest term for obesity‐related metabolic syndrome. The medical world has shifted from non‐alcoholic fatty liver disease (NAFLD) to metabolic‐dysfunction‐associated steatotic liver disease (MASLD), which refers to a group of liver disorders distinguished by hepatic steatosis in the absence of considerable alcohol usage. However, most of the current NAFLD data may still be used for MASLD [64, 65]. MASLD is the major cause of liver disease and is connected with great liver morbidity and mortality. MASLD is estimated to affect 30% of adults in the whole world, and its prevalence increased from 22% to 37% between 1991 and 2019 [66, 67]. The increase in overweight and obesity has been connected with a rise in the occurrence of MASLD. A higher likelihood of fibrosis development is associated with metabolic dysfunction‐associated steatohepatitis (MASH), a more critical form of MASLD characterized histologically by lobular inflammation and ballooning degeneration of hepatocyte cells [70]. In a prospective study in a cohort of MASLD patients who underwent primary liver biopsy, the occurrence of liver‐associated events was 0.43 per 100 person‐years and the heart attack rate was 2.03 per 100 person‐years. Liver‐associated events were observed only in patients with advanced liver fibrosis (combined incidence: 9.1% in patients with advanced liver fibrosis and 9.1% in other patients, and 0% of patients without liver fibrosis). The liver‐related incidence per 100 person‐years in patients having F3 and F4 fibrosis was 1.47 and 3.85, respectively (fibrosis stage ≥ F3 is considered advanced fibrosis; F3 is liver bridging fibrosis while F4 is cirrhosis). The prevalence of MASLD and related complications, including HCC, is expected to increase in later years [69, 70, 71]. A comprehensive systematic review published in 2024 examined the association between MASLD and the incidence of hepatic malignancies, with an emphasis on the impact of fibrosis and MASLD severity as cancer risk factors. The systematic review found an association between MASLD and an increased risk of hepatic cancer. They concluded that the stage of fibrosis in MASLD is a significant independent predictor of mortality and liver‐related events, with higher fibrosis stages indicating a higher risk. Longitudinal data revealed that higher FIB‐4 scores were associated with an increased risk of developing HCC and cirrhosis [74].

A study conducted in South Korea evaluated the clinical outcomes and survival of HBV‐HCC and NAFLD‐HCC. Surgical resection was performed in 232 patients, 200 with HBV + HCC and 32 with NAFLD + HCC. Before propensity score matching (PSM), the mean tumor size in the NAFLD + HCC group was higher than in the HBV + HCC group (4.4 ± 3.3 cm vs. 3.4 ± 1.8 cm, *p* = 0.01 4), and cirrhosis was again greater in HBV + HCC patients (55% vs. 15%, *p* < 0.001). The 5 year overall survival after PSM was comparable among two groups (60% vs. 63%, *p* = 0.978) [75]. Similar to the situation in europe, South Korea and Southeast Asia over the past 20 years, NAFLD is currently the leading cause of HCC among liver transplant patients and waiting lists for liver transplantation in US [74, 75, 76]. There is compelling evidence that older age is independently related with the development of NAFLD as well as the risk of hepatocellular carcinoma in NAFLD patients. Men and women have the same risk of NASH, while women had a 37% greater chance of advanced fibrosis. Women are 56% more likely than males to have advanced fibrosis when they are 50 years or older. Hispanic and Latino persons are more likely to acquire NAFLD than non‐Hispanic Whites [79].

#### 3.5.2 Diabetes

Diabetes is connected with HCC and other malignancies [80]. A study comparing people with and without diabetes found that people with diabetes had two times more risk of HCC compared to people without diabetes [81]. Diabetes increases cancer risk through many mechanisms, including the production of pro‐inflammatory cytokines and reactive oxygen species (ROS), which can promote cell proliferation, induce genetic instability, and prevent hepatocyte apoptosis [80]. There is a risk of infection and infection between diabetes and HCC formation [82]. According to a most recent study, diabetes triples the risk of developing HCC in NAFLD [80]. Diabetes is the metabolic risk factor most strongly associated with hepatocellular carcinoma in NAFLD; however, the risk of hepatocellular carcinoma rises gradually as the number of metabolic risk factors increases. Patients with NAFLD, diabetes, obesity, hypertension, and dyslipidemia have a higher risk of hepatocellular carcinoma (HR 3.9; 95% CI 2.2–7.2) compared to those with NAFLD and none or only one of these metabolic risk factors. In multivariate analysis, diabetes was the only metabolic feature that was independently related to hepatocellular carcinoma. Diabetes had an influence on the risk of hepatocellular carcinoma in NAFLD patients who did not have cirrhosis [79].

NAFLD is one of a significant public health problems of worldwide concern. This condition occurs in people whose liver weighs more than 5%–10% due to excess fat. Diabetes raises the risk of cirrhosis and the development of liver disease in patients having NAFLD [83]. A prospective cohort research with 173,643 diabetic patients and 650,620 non‐diabetic patients found that diabetes was substantially linked with NAFLD (hazard ratio [HR], 1.98) and HCC (HR, 2.16). They also discovered that diabetic individuals with over 10 years of follow‐up were at the highest risk [84]. A hospital‐based case–control research comparing 420 HCC patients and 1104 healthy controls discovered that diabetes was more common in HCC patients (aOR, 4.2). Patients with a diabetes duration of 6–10 years or more had an OR of 1.8 and 2.2 for HCC, respectively, compared to those with a diabetes duration of 2–5 years. This implies that the duration of diabetes is connected with an increased risk of developing HCC [85].

Too much sugar causes negative energy of metabolism and provides more material, thus promoting the growth and spread of tumor cells. When exposed to high glucose levels, HCC cells have a malleable mesenchymal phenotype and undergo metabolic reprogramming through c‐Met activation, thus promoting HCC proliferation and invasiveness [86]. Reactive oxygen species (ROS) are produced in response to high glucose stimulation. ROS can stimulate the liver and promote mitochondrial dysfunction, ultimately leading to cancer and liver damage. In sorafenib‐resistant HCC cells, reduced ROS production has been shown to promote tumor growth [87]. Additionally, a high level of glucose in the blood leads to the accumulation of high levels of glycolytic products. These factors may interact with ROS to form a feedback loop that promotes HCC development through modulation of angiogenesis and proliferation [86, 87]. A nationwide study including 119,316 individuals in two large groups found that hyperinsulinemia and IR were connected with a higher risk of HCC after 25.6 years of follow‐up [90]. Much evidence shows that type 2 diabetes (T2DM) affects the development and spread of HCC [90]. A comprehensive study found that type 2 diabetes was related to a 2.5‐fold increase in the incidence of HCC. Furthermore, the risk estimate from 13 case–control studies revealed a 2.5‐fold increase in the incidence of diabetes in HCC patients compared to controls without diabetes [91].

### 3.6 Intestinal Microbiota

The intestinal microbiota is thought to be particularly important in various gastrointestinal, pancreatic, and liver diseases. Interest in its role in liver carcinogenesis continues to grow, especially in the development of therapeutic strategies. Several studies have shown an association between certain types of cancer and liver cancer, such as Hepatocellular Carcinoma (HCC). Given the stomach‐liver axis, HCC presents a truly interesting area for further research [92]. Recent studies suggest that the stomach and liver are closely related, known as the “stomach liver axis,” and that intestinal bacteria may play a role in keeping a healthy body clean. Additionally, since approximately 70% of liver blood is produced by the intestines, the liver's function as the first line of protection against intestinal antigens is also supported. The finding that NAFLD/NASH is a side effect of jejunoileal bypass surgery for inflammatory bowel disease (IBO) and obesity and diverticulosis supports the association between NAFLD/NASH and intestinal changes—higher axis [93]. Recent research has shown that the stability of the gut microbiota and its metabolites is critical for the preservation of normal liver functioning [83, 84].

In recent years, the possibility that the gut microbiota interacts with the cancer process has received increasing attention. Additional evidence suggests an association between gut microbiota and some malignancies, particularly colon cancer [96]. In recent years, as the understanding of intestinal flora in carcinogenesis and cancer treatment has grown, research has indicated that intestinal flora imbalance is directly associated with HCC [86, 87]. A study published in 2023 revealed that the gut microbiota is crucial in the pathogenesis of HCC and in oncotherapy [99]. Chronic hepatitis exposure to gut microbiota dysbiosis and metabolites leads to cirrhosis and, eventually, hepatocarcinogenesis [89, 90, 91]. Through the portal vein or biliary system, intestinal microorganisms, intestinal microbial‐derived metabolites, intestinal cellular components (peptidoglycans, flagellin, lipoteichoic acid, and lipopolysaccharide), and intestinal epithelial cell exosomes are transported into the liver. They interact with intrahepatic immune cells, causing an inflammatory immune response and causing the normal liver to progress from pre‐cirrhosis to cirrhosis or liver cancer [103].

The liver and intestines are anatomically connected with each other directly by the portal vein. This is what the term “gut‐ liver axis” refers to [96]. Through a special gut‐liver axis, the billions of commensal bacteria that reside in the gut lumen have a significant impact on the physiology and pathology of the liver. Alterations in microbial metabolism, reduced intestinal barrier function, and the translocation of microbial components to the liver can result from disturbances in the gut microbial populations caused by both genetic and environmental causes. These changes work together to cause liver disease, and their ongoing effects over the duration of the illness are crucial for the development of hepatocarcinogenesis. The gut‐derived intrahepatic microbiota also shows up as a new factor in the initiation and spread of liver cancer [104]. Through intricate relationships between the liver, intestine, and gut bacteria, the gut‐liver axis preserves health. In the onset and progression of liver disorders, the gut microbiota plays a far more important role than was first thought. Via a complicated web of interactions in the gut‐liver axis, disturbances in gut homeostasis may particularly cause the liver to become inflamed and fibrogenic. For the host immune system to produce more pro‐inflammatory cytokines, microbial products like lipopolysaccharides (LPS) or other pathogen‐associated molecular patterns (PAMPs) or microbe‐associated molecular patterns (MAMPs) must be present. Initiating a series of immunological reactions in the liver, they can be identified by pattern recognition receptors (PRRs) present on the surface of gut cells or cells in portal circulation. As a result, changes in gut microorganisms are linked to a number of liver disorders and, occasionally, distinct types of cancer. Numerous recent research studies have shown that people with liver disorders such as cirrhosis, MASH, MAFLD, HCC, and iCCA have different gut microbiota profiles [94, 95]. A study found that the degree of gut microbial dysbiosis was linked to the progression of HCC, and that the dysbiosis tended to increase as HCC progressed. Although the alpha diversity of the microbiota differed significantly between the advanced stages of HCC (stage III and IV primary HCC) and healthy controls, the difference between early HCC and healthy controls was less noticeable. However, when compared to patients with liver cirrhosis, the diversity of microbes in HCC patients increases. Proteobacteria were also significantly higher in patients with stages II and III primary HCC than in healthy controls, but Firmicute relative abundances were not significantly altered in patients with primary HCC [107]. It is interesting to note that many probiotic bacteria originate from Firmicutes, but the majority of pro‐inflammatory bacteria are found in Proteobacteria [108]. This finding suggests that pro‐inflammatory microorganisms coexist with primary HCC development. Many pro‐inflammatory bacteria of Proteobacteria, including those of Enterobacteriaceae, also operate as dysbiosis markers at the same time. In patients with stage I HCC, high‐quality sequencing revealed that Actinomyces, Atopobium, Desulfococcus, Enterobacter, Paraprevotella, Planctomycetes, Prevotella, Veillonella, and other unidentified taxa were more abundant than in healthy controls. Patients with stage II HCC showed enrichment in Desulfococcus, Enterobacter, Lactococcus, Leptotrichia, Paraprevotella, Planctomycetes, Prevotella, Veillonella, and other unknown species. Patients with stage III HCC had increased numbers of various unidentified species, including Actinomyces, Atopobium, Desulfococcus, Enterobacter, Haemophilus, Lactococcus, Leptotrichia, Neisseria, Oribacterium, Prevotella, Rothia, Selenomonas, and Veillonella. The number of Desulfococcus, Enterobacter, Prevotella, Veillonella, and several other unidentified species rose across all HCC stages. On the other hand, patients with stage I HCC had lower levels of Acidaminococcus, Cetobacterium, Coprobacillus, Pyramidobacter, Turicibacter, and two unidentified genera, whereas patients with stage II HCC had lower levels of Anaerotruncus, Cetobacterium, and an unidentified genus. Acidaminococcus, Anaerostipes, Anaerotruncus, Butyricimonas, Cetobacterium, Cloacibacillus, Coprobacillus, Holdemania, Methanobrevibacter, Odoribacter, Pyramidobacter, Turicibacter, and four other genera were also decreased in individuals with stage III HCC. The amount of cetobacterium decreased at every stage of primary HCC. Regarding the changes in the gut microbiota composition between HCC patients and healthy individuals, one study notes that both dysbiosis and Proteobacteria are elevated in HCC patients [107]. The gut microbiota profiles of individuals with HCC differ from those of healthy individuals, according to a number of other studies. The primary difference is the rise in *Escherichia coli* , Neisseria, Klebsiella, and other bacteria, and the fall in Firmicutes, Clostridium, and Bifidobacterium, among others [96, 98, 99].

The blood coming towards the liver is much richer in nutrients that are absorbed by the intestines. These compounds can use the pattern to recognize receptors that will trigger an inflammatory response. Chronic inflammation results from changes in the gut microbiota in chronic lung disease, which is connected with damage to the intestinal lining. As the condition progresses, fibrosis and tissue repair may occur. Rethinking the source of pain leads to the possibility that HCC is the final stage of the entire disease process [100, 101]. Bacterial products activate toll‐like receptors (TLRs) in the liver called TLR 4. This initiates the NF kB pathway, which initiates mitotic signaling connected with the stoppage of programmed cell death. Due to chronic damage, the liver is affected by chronic activation of various TLR ligands and other chemical agents. These drugs act as inflammatory mediators, making liver cells more resistant and underpinning the development of HCC [113].

## 4 Other Potential Risk Factors

One of the significant risk factors for HCC is pollution. In 2022, a cross‐sectional study included 348 patients having chronic hepatitis C who were examined for HBsAg, HBcIgG, anti‐HCV, and HBsB. The International Classification of Diseases Tenth Revision (ICD 10) is the basis for HCC diagnosis. Daily estimates of air pollution were combined by date of recruitment or HCC diagnosis to obtain an average estimate for the previous year. Twelve of the 348 patients (3.4%) had HCC. Individuals diagnosed with HCC showed significant differences; they were older (71.7 years vs. 50.9 years; *p* = 0.004), had higher HBsAg seroprevalence (41.7% vs. 5.1%; *p* < 0.001), and were older than 2.5 years. They had higher levels of elements (PM2.5) (21.5 vs. 18.2 μg/m^3^; *p* = 0.05). Logistic regression analysis showed that the following variables were affected by HCC: age (OR: 1.10; CI, 1.03–1.17; *p* = 0.01), PM2.5 level (OR: 1.51; CI, 1.02–2.0; *p* = 0.04), and was associated with HBsAg seropositivity (OR: 6.60; CI, 1.51–28.85; *p* = 0.01). The likelihood of developing HCC was affected by HBsAg seropositivity and PM2.5 (OR: 22.17; CI, 3.33–147.45; *p* = 0.001). Once hepatitis C is controlled, PM2.5 is still a major risk factor for HCC. Further studies are still required to determine long‐term patient outcomes [114]. Other studies on the development of HCC have shown that it leads to changes in hemochromatosis, α1‐antitrypsin deficiency (SERPINA1), glycogen storage disease (G6PC, SLC37A4), tyrosinemia (FAH), porphyria (HMBS, UROD), and Wilson disease (ATP7B). A sum of 242 individuals with HBV‐associated LC were enrolled and followed up in another study. Logistic regression analysis was performed in order to understand HCC risk factors. The average follow‐up period was 37 months (range: 6–123 months). By the end of the analysis, HCC was detected in 11 patients (11.3%) with compensated cirrhosis (CC) and 45 patients (31.0%) with decompensated cirrhosis (DC). The TyG index of the HCC group was higher compared to the non‐HCC group (*p* = 0.05). The TyG index has been observed to be a significant risk factor for HCC in LC patients [115]. Hereditary hemochromatosis causes an approximately 100–200 times higher incidence of HCC.

### 4.1 Hereditary Hemochromatosis

According to recent research, male patients with cirrhosis and a homozygous C282Y mutation who have significant iron overload are most at risk for developing hemochromatosis‐related cancer (HCC) [116]. For HCC, hereditary hemochromatosis (HH) is a significant genetic risk factor. The hallmark of HH, an autosomal recessive condition of iron metabolism, is increased iron accumulation in the liver and most other organs, which eventually results in organ failure. One of the complications of HH that almost invariably affects patients with cirrhosis and raises death rates is HCC [117]. Because hemochromatosis causes an iron excess, underlying processes like enhanced cell proliferation, peroxidase‐induced damage to DNA and cell membranes, and elevated ROS levels all contribute to the formation of tumors [118]. About 8%–10% of HH patients have HCC, while 10%–25% of HH patients have cirrhosis [119]. Despite the incomplete understanding of the HFE gene's molecular and physiological roles in the liver, a number of case–control and population‐based studies have demonstrated that HFE mutations increase the chance of developing HCC [109, 110]. Studies based on populations offer a more accurate estimate of the general incidence and prevalence of HCC in HH. According to research conducted by the US National Centre for Health Statistics, HH and HCC are closely related. In this study, the odds of having HCC were 23 times higher for patients with HH who passed away than for those who did not have HH [122]. Another study in Sweden found that HH people had a roughly 20‐fold increased risk of HCC compared to the general population. HH males had a 6% absolute risk of HCC at 10 years of follow‐up, greater than the risk for women (1.5%) [123]. A study discovered that the C282Y homozygous mutation was present in 7% of HCC patients [124]. A related study discovered that eight of the 118 C282Y homozygotes—or 1.8% of the patients with HCC—developed HCC [125]. A meta‐analysis was performed to investigate the correlations between HCC and C282Y and H63D mutations. It was shown that C282Y homozygotes (YY vs. CC) had an odds ratio of 11 for the incidence of HCC [126]. An additional analysis of 43 published studies (5758 cases and 14,741 controls) showed that the homozygous mutation for HFE C282Y was substantially linked to a higher risk of HCC in comparison to the general population [127].

### 4.2 Tobacco Consumption (Smoking)

A comprehensive review of 81 epidemiological researches found a connection between smoking and a higher risk of HCC morbidity and mortality [128]. Heavy smoking can cause excess iron to enter the liver, leading to fibrosis and cancer. There is also evidence of a reduction in the P53 gene [129]. A study in the central delta region of Egypt revealed that these two behaviors are non‐HBV and non‐HCV risk factors due to several factors [130]. In Europe, a nested case–control research revealed that smoking was linked to 47.6% of HCC cases [131]. In 2004, the IARC (International Agency for Research on Cancer) determined that there was sufficient evidence to designate liver cancer as a tobacco‐related malignancy [132]. Most of what is now known about the connection between tobacco use and various malignancies is derived from epidemiological data. In comparison to non‐smokers, present and past smokers had substantial pooled ORs for HCC development of 1.55 (95% CI, 1.46–1.65) and 1.39 (95% CI, 1.26–1.52), respectively, according to the results of a meta‐analysis of 81 epidemiological studies. Additionally, a dose–response effect was found, showing that heavy smokers had a significantly higher risk of liver cancer (OR 1.9; 95% CI, 1.68–2.14) [128]. According to the carcinogenic potential of several compounds with oncogenic potential, such as 4‐aminobiphenyl, a liver carcinogen that has been identified as a causative risk factor for liver cancer, smoking may have an effect on the development of HCC and have a biological substrate. By triggering miscoding events in important genes, these carcinogenic substances covalently attach to DNA to form DNA adducts, which play a crucial part in carcinogenesis [133]. Smoking raises pro‐inflammatory cytokine production and oxidative stress, both of which contribute to inflammation [134].

### 4.3 Chemical Compounds Causing HCC

Two types of drugs have been applied in the development of HCC, organic drugs and inorganic drugs. Cadmium and arsenic are two weak elements [135]. N‐nitrosamines, PCBs, PCB monomers, polyvinyl chloride, perchloroethylene, trichloroethylene, dioxin‐like compounds, and polybrominated biphenyls are examples of organic compounds. Other substances considered to be risky include chemicals such as dichlorodiphenyltrichloroethane (DDT), otoluidine, and chloral hydrate used in pesticides and herbicides [136]. According to the International Association of Cancer Registries (IACR), arsenic is considered a Group 1 carcinogen. Various epidemiological researches have shown that long‐term exposure to arsenic can cause preneoplastic diseases, liver dysfunction, hepatomegaly, cirrhosis, and liver fibrosis [137]. Vinyl chloride, which is used to make PVC plastics, can lead to hepatocellular carcinoma, uncommon liver angiosarcoma, and other cancers. The hepatocarcinogenic metals arsenic and cadmium are found naturally and are highly exposed at work in businesses that use coal, metals, plastics, and batteries. Millions of workers in manufacturing and waste disposal are exposed to organic solvents and N‐nitrosamines, which have IARC classifications ranging from potentially carcinogenic (group 2B) to carcinogenic (group 1). Although the insecticide DDT may cause hepatocarcinogenesis (group 2B), it is nevertheless used to prevent malaria in underdeveloped nations [138]. Polyvinyl chloride (PVC), a crucial ingredient in the plastics industry, is made from vinyl chloride monomer (VCM), a hydrocarbon. Water pipes, window frames, insulation, waterproof clothing, and dental and medical equipment are all made of PVC, which is a safe material. Nonetheless, VCM, the monomeric and reactive form of vinyl chloride, has been exposed to thousands of workers engaged in the plastics manufacturing process. Additionally, cigarette smoke contains VCM. To evaluate occupational exposure, the VCM metabolite thioglycolic acid is found in the urine [139]. VCM toxicity has been shown to disrupt the liver endothelium, leading to portal hypertension and the uncommon cancer known as angiosarcoma of the liver (ASL). Only VCM exposure is linked to the proliferation of endothelial cells known as ASL. Additionally, VCM has been demonstrated to have a synergistic impact with alcohol, increasing the risk of cirrhosis, HCC, and total liver cancer mortality [140]. VCM is inhaled and then converted by the liver into a variety of mutagenic and carcinogenic substances, including chloroethylene oxide and ethylene dichloride [130, 131]. These byproducts, which are mostly concentrated in the liver where processing takes place, include carbamates, which cause chromosomal abnormalities and DNA breakage [143]. KRAS and p53 oncogenic mutations have been found in workers with HCC exposed to VCM [142]. Heavy metal arsenic (As) has been designated as a category 1 carcinogen by the IARC. Inorganic arsenic seeping into groundwater is the main exposure source. Bangladesh, West Bengal, and the Northern Chilean province of Antofagasta are among the places in the globe where as is very prevalent in groundwater [144]. Glutathione in the liver detoxifies arsenic once it is absorbed through the digestive system. Alternatively, glutathione can be converted to arsenic and eliminated in the bile, or it can function as an antioxidant. The liver also methylates arsenic [145]. Numerous epidemiological investigations have demonstrated a link between exposure and a variety of hepatic conditions, such as liver cancer, cirrhosis, fibrosis, sclerosis, and hepatomegaly [146]. Since arsenic is known to build up in the liver, a number of carcinogenesis mechanisms have been proposed, including direct cytotoxicity, genotoxicity, oxidative stress and free radical generation, disruption of signal transduction and cellular proliferation, and significant changes in DNA methylation [136, 137]. Originally identified as a yellow/orange color, cadmium (Cd) is a heavy metal that occurs naturally as a gray‐white powder. It is used as an anti‐corrosive veneer for steel and iron and has subsequently become a crucial component of nickel‐cadmium rechargeable batteries. It has been demonstrated that cadmium builds up in the liver, lungs, kidneys, and pancreas and that it negatively affects these organs as well as the blood, immunological system, and brain system in animals [149]. Studies on animals have demonstrated that Cd can cause hepatocarcinogenesis through a variety of mechanisms. In cell and animal models, Cd has been implicated in various stages of carcinogenesis, such as oxidative stress‐induced mutation generation, Kupffer cell‐induced inflammatory IL‐6, protooncogene activation through DNA hypomethylation, impaired DNA repair, and E‐cadherin dysfunction, which promotes metastasis [150]. A cross‐sectional study from China that looked at aflatoxins in people discovered a link between HCC mortality and dietary consumption of Cd [151]. Numerous epidemiological studies have revealed that pesticides raise the incidence of HCC in agricultural workers, and numerous widely used chemicals have been shown to cause cancer in animal models. Nevertheless, the only particular pesticide that has been studied and linked to human liver cancer is 1,1,1‐Trichloro‐2,2‐bis(p‐chlorophenyl)‐ethane (DDT) [152]. In animal models, DDT has been shown to cause carcinogenesis and liver damage [153]. In light of these findings, the IARC classified DDT as a potential human carcinogen (category 2B) [154]. Per‐ and polyfluoroalkyl compounds (PFAS), which are quite prevalent and can harm the liver, refer to as “forever chemicals”. They are difficult to break down and can remain in the body for years. High exposure to perfluorooctanesulfonic acid, one of the most prevalent PFAS compounds, was shown to be associated with a higher risk of hepatocellular carcinoma in people. In a case–control study, 50 incident HCC patients and 50 individually matched controls from the Multiethnic Cohort (MEC) cohort had their pre‐diagnostic plasma PFAS and metabolomics evaluated. The study location, sex, age, and race were used to match the cases and controls. Using conditional logistic regression, the relationship between PFAS exposure and HCC risk was investigated. Key metabolites and metabolic pathways were identified using a meet‐in‐the‐middle strategy, and a metabolome‐wide association study and pathway enrichment analysis were conducted for PFAS exposure and HCC risk. According to the results, a 4.5‐fold higher risk of HCC was linked to high levels of perfluorooctane sulfonic acid (PFOS) (90th percentile from NHANES; > 55 μg/L) (odds ratio 4.5, 95% CI 1.2–16.0). According to pathway enrichment analysis, exposure to PFOS was linked to changes in the pathways for the production of amino acids and glycans, which were likewise linked to an increased risk of HCC. Four metabolites were found to be positively correlated with PFOS exposure and HCC risk: glucose, butyric acid (a short‐chain fatty acid), α‐ketoisovaleric acid (a branched‐chain α‐keto acid), and 7α‐hydroxy‐3‐oxo‐4‐cholestenoate (a bile acid) [155].

### 4.4 Gender Difference

Approximately 80% of initial liver tumors are HCC. Men are two to three times more likely than women to have it, depending on the regions. Some European nations (such as France and Malta, where the male to female ratio is 5.0 and 4.8, respectively) have the biggest disparity, while other nations (like Uganda, Costa Rica, Ecuador, and Colombia) have little differences, up to an equal ratio between the sexes [156].

The incidence of HCC varies by gender. It has the ninth highest incidence in women (3.4%) and the fifth highest incidence in men (7.5%) [137]. Among newly diagnosed malignancies in 2020, liver cancer accounted for 3.0% (273,357 cases) in women and 6.3% (632,320 cases) in men. Typically, HCC strikes men earlier than it does women. Specifically, HCC formation probably happens in men between the ages of 50 and 69, whereas in women, the incidence seems to be comparable between the ages of 50 and 69 and aged 70 and above [157]. Women are less prone to liver damage because estrogen plays a very important role in suppressing IL‐6 mediated inflammation. Estrogens have a preventive function in women, preventing the growth of HCC and reducing hepatic fibrogenesis and inflammation. Furthermore, they are partly responsible for liver cancer's decreased aggressiveness, improved responsiveness to therapy, decreased recurrence rates, and generally improved prognosis [158]. An important factor affecting hepatocyte growth in men is the enhancement of androgen receptor signaling by testosterone. Androgens increase the chance of developing HCC by promoting hepatocarcinogenesis and cell proliferation [159].

### 4.5 Obesity

Obesity is a chronic, relapsing, and progressive condition marked by excessive body fat. The World Obesity Federation reported that over 700 million individuals, or over 15% of the global population, were obese in 2020. By 2030, this figure is expected to exceed one billion persons [160]. Obesity is most commonly diagnosed clinically using the body mass index (BMI), which is the ratio of body weight to height squared (kg/m^2^). Severity is divided into three classes: class 1 (30–34.9 kg/m^2^), class 2 (35–39.9 kg/m^2^), and class 3 (≥ 40 kg/m2) [161]. Obesity increases the risk for cancer for various reasons. Obesity has lately been linked to an increased risk of at least 13 forms of cancer, including meningioma, esophageal adenocarcinoma, multiple myeloma, kidney, uterine, ovarian, thyroid, breast, gallbladder, stomach, pancreas, colorectal, and liver cancer [162]. Obesity and HCC have a unique association in that they share a known, direct link via MAFLD. Obesity is the leading cause of MAFLD. MAFLD is as common as obesity, affecting around 30% of the population [69]. In obesity, steatosis happens when the pace at which fatty acids emerge surpasses the liver's ability to use and eliminate them, leading to net retention in the form of cholesterol, triglycerides, and/or lipid droplets. The exact diagnostic criteria defining excessive steatosis are still unknown, despite the general agreement that a certain amount of hepatic fat is good. The current criterion for diagnosing hepatic steatosis is intrahepatic fat ≥ 5% of total tissue mass [163]. Other pathophysiological features of obesity and metabolic syndrome, such as dyslipidemia and persistent low‐grade inflammation, may make it possible for HCC to develop directly without first developing cirrhosis. Both the synthesis of new cholesterol and the absorption of extra cholesterol in the bloodstream are carried out by the liver. This may result in oxidized LDL buildup in the setting of dyslipidemia, which can promote HCC by causing inflammation and lipotoxicity [153, 154, 155].

Many studies have shown that obesity is connected with insulin resistance and increased levels of insulin‐like growth factor, which acts as mitogens that promote cell growth. A meta‐analysis found obesity itself increases the risk for liver cancer. Of the 11 clinical studies, 7 included obese people (*n* = 5037) and 10 included obese people (*n* = 6042) [167]. Compared with normal‐weight individuals, the associated risk for HCC in obese individuals was 1.17 (95% CI: 1.02–1.34) and in obese individuals, it was 1.89 (95% CI: 1.51–2.36) [167]. Another study included 25,337 patients with hepatocellular carcinoma (HCC) in 26 prospective studies. This association continues regardless of geographical location and gender. Although the incidence is higher in men as compared to women, this difference may be due to differences in fat, such as a higher incidence of visceral fat in men [168].

### 4.6 Oral Contraceptive Pills

Interestingly, oral contraceptive use for an average of 11 years can cause liver adenomas. This has led to debate about the effects of hormones on HCC development [169]. Effects of estrogen intake on the role of sex hormones in the assessment of HCC. Oral contraceptive use has been shown to be a good predictor for HCC patients [170]. There is some scientific and experimental evidence that oral contraceptives may have a role in hepatocarcinogenesis. Estrogen has been shown in in vitro trials to increase the rate of spontaneous mutation and the cellular proliferation of HepG2 cells [160, 161]. Patients who used oral contraceptive pills with more than 50 μg of estrogen had a higher risk of liver illness linked to oral contraceptive usage, and their condition improved once they stopped using the contraceptive [1, 2]. All of these results point to the possibility of hepatocarcinogenic consequences from the use of contraceptives, which might vary depending on a number of variables such as exposure duration, dose levels, and the impact of confounding variables [3].

### 4.7 AIH

In observational studies, autoimmune hepatitis (AIH) seemed to be an uncommon cause of HCC. After the hepatitis C virus (HCV) was discovered in 1989, it could have been even more uncommon. In 1988, it was found that 7% of AIH patients with cirrhosis that had lasted at least 5 years had HCC [173]. After ruling out a persistent HCV infection, the same researchers discovered in 2000 that just 1% of HCC patients had cirrhosis linked to AIH [174]. According to estimates from 2013, the annual prevalence of HCC in patients with cirrhosis was 1.1%–1.9%, while the frequency in patients with AIH and cirrhosis was between 1% and 9% [174]. Despite AIH predominating in female patients, HCC developed in the same percentage in male and female AIH patients [164, 165]. According to estimates from 2015, the incidence of HCC in patients with AIH ranged from 0.45% to 0.64% in the Asia Pacific area and from 0.56% to 1.9% in Europe and America [177]. A review of 11 studies found that 5%–6% of autoimmune hepatitis (AIH) patients developed hepatocellular carcinoma (HCC), with the majority of cirrhosis in AIH patients ranging from 12% to 83% [178]. However, further research is needed to clarify the link between AIH and HCC incidence.

### 4.8 Alpha 1 Antitrypsin Deficiency (A1ATD)

Alpha‐1 antitrypsin is produced in the liver and subsequently released into the bloodstream, where it makes its way to the lungs, where it performs its primary role. The molecule cannot exit the liver in alpha‐1 antitrypsin deficiency (A1ATD), and instead builds up in the liver cells, resulting in cirrhosis, liver fibrosis, and perhaps hepatocellular carcinoma (HCC) [179]. Alpha‐1 antitrypsin deficiency (A1ATD) occurs concurrently with other risk factors such as liver disease, NAFLD, and HCV; A1ATD‐associated HCC is connected with several mechanisms, including mutations in cyclin D1 and melanoma cell adhesion molecule (MCAM) regulation, delayed endoplasmic reticulum protein degradation, and mitochondrial dysfunction [180] (Tables 2 and 3).

**TABLE 2:** Risk factors' classification involved in the development process of HCC [181].

Category	Risk factors identified for HCC
Infectious	Hepatitis B virus (HBV), Hepatitis c virus (HCV)
Non infectious	Non‐infectious factors include modifiable medications, modifiable alcohol consumpt ion, and modifiable cigarette use
Risk factors related to host	Nonmodifiable factors included gender, race, and autoimmune disease. Regulation: oral contraceptives, obesity, diabetes, NAFLD
Monogenic (single gene) risk factors	It includes Alpha 1 antitrypsin deficiency and hemochromatosis
Polygenic (Multiple genes)	It includes Family history and aflatoxins

Abbreviations: HBV, hepatitis B virus; HCV, hepatitis c virus; NAFLD, Non alcoholic fatty liver disease.

**TABLE 3:** Gender disparities in HCC risk factors, clinical characteristics, and therapy response.

	Risk factors	Male	Female
Risk factor's	HBV	High	Low
	HCV	Low	High
	Alcohol	High	Low
	Obesity	Low	High
Prevalence	T2DM	High	Low
	PBC/AIH	Low	High
	Smoke	High	Low
Clinical features	Occurrence	High	Low
	Development age	Early	Late
	Size (during diagnosis)	Large	Small
	Encapsulation ratio (during diagnosis)	Low	High
	Multifocality ratio (during diagnosis)	High	Low
	Vascular invasion incidence (during diagnosis)	High	Low
	Metastasis ratio (during diagnosis)	High	Low
	Overall stage condition (during diagnosis)	Most advance	More Early
	Course way	More aggressive	Less aggressive
	Prognosis condition	Bad	Better
Response to treatment	Response towards treatment	Worst	Better
	Recurrence ratio	High	Low

Abbreviations: AIH, Autoimmune hepatitis; HBV, hepatitis B virus; HCV, hepatitis C virus; T2DM: type 2 diabetes mellitus.

## 5 Surveillance for Individuals at High Risk

Those who are at a high risk of developing HCC are advised to undergo surveillance [182]. International guidelines recommend that the following groups be regularly monitored: patients with cirrhosis of any cause, with the exception of those with Child‐Pugh class C cirrhosis who are not waiting for a liver transplant; people with chronic HBV infection, even if they do not have cirrhosis, in specific subgroups, such as males aged at least 40, females aged at least 50, and patients with a family history of HCC at any age; and people with chronic HCV infection who have advanced fibrosis (Metavir stage F3) without cirrhosis. There is little data on the prevalence of HCC in individuals with non‐viral chronic liver illnesses that do not include cirrhosis, such as Wilson's disease, autoimmune liver disease, hereditary hemochromatosis, alcohol‐associated steatohepatitis and MASH, and alpha‐1 antitrypsin deficiency. To promote early identification and enhance survival, hepatic ultrasonography every 6 months is the suggested monitoring method, whether or not serum alpha‐fetoprotein (AFP) is measured [183]. Compared to those who did not get surveillance, studies have shown that surveillance results in a lower death rate, greater eligibility for curative therapies, and earlier diagnosis of HCC [184].

## 6 Primary Prevention Strategies

One of the main causes of cancer‐related death globally is hepatocellular carcinoma (HCC), which usually arises as a result of chronic liver illness. Geographical differences exist in the incidence and mortality rates of HCC; East Asia and sub‐Saharan Africa have been found to have the greatest incidences [185]. The high incidence of chronic hepatitis B virus (HBV) infection in these areas is primarily responsible for this difference [186]. At over 50% of cases globally, chronic HBV infection continues to be a major etiological cause for HCC [187]. The incidence of HCC, on the other hand, has been increasing in Western nations because of conditions like alcohol‐associated liver disease (ALD), metabolic dysfunction‐associated steatotic liver disease (MASLD), formerly known as nonalcoholic fatty liver disease (NAFLD), and chronic hepatitis C virus (HCV) infection [11, 177]. The preventative strategies of HCC that we will examine below demonstrate that the incidence of HCC is greatly reduced by hepatitis B vaccination and antiviral therapies for hepatitis B and C. The risk of HCC can be decreased by making lifestyle changes, such as cutting back on drinking, quitting smoking, and keeping a healthy weight through diet and exercise. Prevention is also aided by environmental controls that reduce exposure to aflatoxins and other dangers. Frequent monitoring of high‐risk populations increases survival rates and allows for early identification.

### 6.1 Vaccination Programs

#### 6.1.1 Hepatitis B Virus Vaccination

One of the best primary preventative strategies for HCC is HBV vaccination. Particularly in endemic areas, universal HBV vaccination programs have dramatically decreased the incidence of HBV‐related HCC and the prevalence of HBV infection [189]. A historic study conducted in Taiwan showed that once a statewide HBV vaccination program was put in place, the incidence of HCC in children significantly dropped [190]. The World Health Organization (WHO) advises that in order to reduce mother‐to‐child transmission (MTCT) worldwide, all newborns should have the HBV vaccination within 24 h after delivery, followed by at least two further doses [191]. Consequently, HBV vaccination continues to be a crucial part of international health initiatives meant to lessen the incidence of HCC.

#### 6.1.2 Vaccine Development for Hepatitis C Virus

The hepatitis C virus (HCV) has no approved vaccine at this time because of the virus's tremendous genetic diversity and the intricate immune responses needed for defense [192]. To address these obstacles, current research attempts to create an HCV vaccine that effectively stimulates a robust cellular immune response and a wide range of neutralizing antibodies. By halting the course of liver disease and chronic infection, such a vaccine might drastically lower the number of new infections and, in turn, HCV‐related hepatocellular carcinoma (HCC) occurrences [193]. In a recent phase 1–2 randomized, double‐blind, placebo‐controlled study, persons at risk for HCV infection were given an HCV vaccination regimen consisting of a recombinant chimpanzee adenovirus 3 vector and a booster shot with modified vaccinia Ankara. The vaccination did not significantly prevent chronic HCV infection as compared to a placebo, despite being safe, inducing HCV‐specific T‐cell responses, and lowering peak HCV RNA levels [194].

### 6.2 Lifestyle Modifications

#### 6.2.1 Reduction in Alcohol Consumption

As chronic alcohol use causes liver cirrhosis and encourages carcinogenesis through mechanisms like oxidative stress, inflammation, and compromised immune surveillance, it is a known risk factor for HCC [195]. Reducing alcohol use has been shown to reduce the risk of HCC, especially in communities with high ALD prevalence [196]. At this time, there is no longer a “safe amount” of alcohol consumption [197]. According to a massive database research including 28 million people worldwide, the amount of alcohol intake that minimizes alcohol‐related damage is zero [198]. Likewise, a 4.9‐year follow‐up study of 58,927 Korean patients with MASLD found that deteriorating fibrosis scores were likewise linked to light and moderate drinkers [199]. Taxation, availability control, and educational campaigns are examples of public health measures that have been successful in lowering alcohol‐related damage and, as a result, the risk of HCC.

#### 6.2.2 Managing Weight and Engaging in Activities

Metabolic syndrome and obesity are linked to MASLD, which can lead to metabolic dysfunction‐associated steatohepatitis (MASH) and raise the risk of HCC [200]. It has been demonstrated that regular exercise and keeping a healthy body weight reduce the risk of developing HCC by reducing hepatic fat buildup, improving insulin sensitivity, and reducing liver inflammation [201]. Significant improvements in fibrosis and steatosis can be attained in people with MASLD by losing at least 7%–10% of their body weight [202]. Additionally, a meta‐analysis revealed that physical exercise has a dose‐dependent effect on lowering the risk and mortality of liver cancer [203]. A minimum of 2 h of physical exercise each week is required to lower the death rate from liver cancer.

#### 6.2.3 Dietary Measures

Dietary treatments are important in the primary prevention of HCC because they modulate risk variables such as obesity, metabolic syndrome, and MASLD while also directly impacting liver carcinogenesis via bioactive chemicals [204]. High‐calorie meals rich in trans and saturated fats, cholesterol, and fructose‐sweetened drinks enhance visceral adiposity and promote hepatic lipid accumulation, which leads to MASLD [205]. A diet high in fruits, vegetables, and whole grains, as recommended by the Mediterranean diet, lowers the risk of HCC because these foods have anti‐inflammatory and antioxidant qualities that lower inflammation and oxidative stress, two major factors in the development of hepatic cancer [206]. Since red and processed meats include hemeiron and carcinogenic chemicals (such as heterocyclic amines and polycyclic aromatic hydrocarbons) that are created during processing and high‐temperature cooking and have been associated with an elevated risk of HCC, it is advised to limit consumption of these foods [207]. Regular coffee and green tea intake has been negatively correlated with HCC, maybe as a result of their antioxidant constituents, which include caffeine and chlorogenic acids, as well as their catechins, which include epigallocatechin gallate (EGCG), which have anti‐inflammatory and anti‐carcinogenic qualities [208]. Fatty fish and some plant oils include omega‐3 fatty acids, which have been demonstrated to prevent the development of HCC by altering liver lipid metabolism, reducing hepatic steatosis, and having anti‐inflammatory and anti‐fibrotic properties [209].

#### 6.2.4 Smoking Cessation

A well‐established independent risk factor for HCC is tobacco use; meta‐analyses have shown that smokers are far more likely to develop HCC than non‐smokers [210]. By inducing oxidative stress, damaging DNA, and encouraging hepatic inflammation and fibrosis, carcinogenic chemicals in tobacco smoke aid in the development of hepatocarcinogenesis [211]. Interestingly, the risk of HCC is almost the same for people who stopped smoking more than 30 years ago as it is for those who never smoked [212].

### 6.3 Environmental Management

#### 6.3.1 Reducing Exposure to Aflatoxin

Aflatoxin B_1_ is a major risk factor for HCC in underdeveloped nations. It is generated by Aspergillus species and is mostly spread through contaminated food [213]. The prevalence of HCC has been successfully decreased by strategies to lower aflatoxin exposure, such as enhancing agricultural practices, making sure food is stored properly, and using processing techniques to avoid fungal contamination. Reducing exposure in impacted communities also requires the implementation of legislation to monitor and manage aflatoxin levels in the food supply [214].

#### 6.3.2 Management of Environmental and Occupational Hazards

An elevated risk of developing HCC has been linked to exposure to industrial chemicals such as vinyl chloride, arsenic, and polychlorinated biphenyls (PCBs) [215]. Strict safety procedures, such as the use of personal protective equipment and appropriate ventilation systems, are necessary in occupational contexts where these carcinogenic compounds are handled in order to reduce exposure. Reducing the incidence of HCC associated with environmental pollutants also requires environmental actions, such as controlling industrial emissions and avoiding soil and water pollution [216].

#### 6.3.3 Management of Genetic and Metabolic Conditions

Genetic conditions such as Wilson's disease, alpha‐1 antitrypsin deficiency, and hereditary hemochromatosis can raise the risk of HCC and cause persistent liver damage [217]. The advancement of the disease can be stopped by early detection through genetic screening and suitable treatment, such as phlebotomy for hemochromatosis [218].

### 6.4 Risk Assesment/Surveillance Tools

The most common cause of cancer‐related death worldwide is hepatocellular carcinoma (HCC). Patients who are at high risk for HCC, such as those with cirrhosis, should be enrolled in surveillance programs that perform ultrasound every 6 months, according to current international guidelines. Numerous studies conducted in recent years have further characterized the usefulness of established screening strategies and have introduced new promising tools for HCC surveillance [219]. Following are the tools used for assessment/Surveillance, which include imaging and circulating biomarkers.

#### 6.4.1 Ultrasound

Numerous worldwide recommendations advocate US imaging as the primary modality for HCC monitoring, and it is the undisputed standard for screening cirrhosis patients [206, 209]. There are several advantages to using the US as a screening method for HCC. It is inexpensive, accessible, and well‐tolerated. Furthermore, the use of the US for HCC surveillance is well supported by evidence [221]. According to a comprehensive analysis of 14 US studies, the pooled estimates of US's sensitivity for detecting HCC were 60% (95% CI 44–76) and 97% (95% CI 95–98) [222]. Another extensive meta‐analysis of 32 studies with 13,367 patients revealed that the US had an overall sensitivity of 84% (95% CI 76%–92%) for HCC at any stage and 47% (95% CI 33%–61%) for HCC at an early stage [221]. According to real‐world data from a retrospective cohort multicenter study of 374 patients, 42% of the HCCs were diagnosed during screening. US detection was linked to better survival (hazard ratio 0.41; 95% CI 0.26–0.65), a higher rate of curative treatment (31% vs. 13%; *p* = 0.02), and an earlier tumor detection rate (63.1 vs. 36.4%; *p* < 0.001) when compared to HCC not detected by screening [223]. All of these studies demonstrate that the US is still a useful technique for screening for HCC.

#### 6.4.2 CT and MRI

Since both MRI and CT have demonstrated excellent sensitivity and specificity for the diagnosis of HCC, their technical superiority may theoretically overcome the limitations of US imaging [213, 214]. The diagnostic sensitivity and specificity of MRI are over 90% for HCC lesions larger than 2 cm in diameter, and they remain quite high (88% and 94%, respectively) per patient for lesions of all sizes [226]. The chance of a lesion being identified as an HCC is evaluated by specific LI‐RADS criteria for MRI or CT. HCC lesions can be diagnosed with radiographic signs such as arterial phase hyperenhancement (sensitivity 65%–98%), washout (sensitivity 50%–79%), and an enveloping pseudocapsule (sensitivity 42%–64%) [227]. A meta‐analysis of 17 studies comprising 2760 patients supported the validity of the LI‐RADS classification, showing that the percentage of HCCs was greater in lesions with higher LI‐RADS category [228].

#### 6.4.3 Alpha‐Fetoprotein

The key serological marker employed in the identification of HCC is AFP, a protein from the albumin family [229]. Using a pathological threshold of 20 ng/mL plasma concentration for diagnosis, elevated AFP can be seen in 60%–80% of HCCs [230]. Crucially, US imaging is typically used in combination with AFP measurements. A meta‐analysis verified that US alone had lower sensitivity rates for early‐stage HCC (RR 0.81; 95% CI 0.71–0.93) and any‐stage HCC (RR 0.88; 95% CI 0.83–0.93) than US plus AFP [221].

#### 6.4.4 AFP‐L3

As previously stated, AFP has a rather low specificity since it can be raised in liver cancers that are not HCC and other chronic inflammatory diseases. By identifying the biomarker AFP‐L3, *lens culinaris* agglutinin‐reactive fraction of AFP, which has been demonstrated to be more precisely related to HCC and to have a greater diagnostic effectiveness when paired with AFP measures, this restriction has been partially resolved [231]. Additionally, even in the absence of increased AFP, a study by Kumada et al. found that elevated AFP‐L3 levels were predictive of early HCC [232]. At present, the Food and Drug Administration (FDA) has authorized AFP‐L3 for the evaluation of liver cancer risk as a component of the GALAD score [233].

#### 6.4.5 Des‐Gamma‐Carboxy Prothrombin

Another serum biomarker for HCC is des‐gamma‐carboxy prothrombin (DCP), also known as protein‐induced by vitamin K absence II. DCP is a prothrombin precursor that has differentially undergone abnormal post‐translational carboxylation in malignant cells, and it has been found to be elevated in patients with HCC. The body of evidence supporting DCP as a potential biomarker for HCC is still growing, as DCP levels are not only correlated with HCC stage and survival, but DCP may be more sensitive than AFP for HCC detection [223, 224]. The FDA has authorized DCP for use in determining HCC risk due to its potential promise [220].

#### 6.4.6 Circulating Tumor Cells

The identification of circulating tumor cells (CTCs) for the diagnosis of HCC has long been of interest [236]. Epithelial cell adhesion molecule (EpCAM)‐positive CTCs have been useful in the diagnosis and prognosis of HCC, according to a few studies [237]. However, because most HCCs do not express EpCAM and there are few CTCs in early‐stage tumors, it is difficult to detect early‐stage tumors, which results in a low sensitivity for CTC detection in HCC. Additionally, it has been reported that CTCs that test positive for TWIST1 (twist family BHLH transcription factor 1), AFP, GPC3, DNA‐dependent protein kinase, and vimentin can be used to identify HCC [227, 228].

#### 6.4.7 Other Circulating Biomarkers for HCC

Glypican‐3 (GPC3), a heparan‐sulfate proteoglycan, is known to be raised in HCC tissues. Although serum GPC3, both the full length and N‐terminal form, has frequently been demonstrated to be higher in individuals with HCC [240]. Another possible biomarker for early HCC detection is golgi protein‐73 (GP73), which has AUROC (0.79–0.94), sensitivities (69%–95%), and specificities (83.9%–92.9%) comparable to AFP [241]. Other promising circulating biomarkers for the early diagnosis of HCC include fibronectin, osteopontin, midkine, Dickkopf‐1, squamous cell carcinoma antigen, and others that require validation in large, multicenter studies involving patients with HCCs from various etiologies [231, 232, 233, 234, 235].

## 7 Conclusion

Hepatocellular carcinoma (HCC) remains a formidable global health challenge, with an evolving spectrum of risk factors that necessitate urgent attention. While chronic viral infections such as hepatitis B (HBV) and hepatitis C (HCV) have historically been the dominant etiological factors, the rising prevalence of metabolic dysfunction‐associated steatotic liver disease (MASLD), obesity, type 2 diabetes mellitus (T2DM), and other metabolic syndromes has reshaped the epidemiological landscape of HCC. Environmental exposures, genetic predispositions, and gut‐liver axis alterations further contribute to disease onset and progression, highlighting the multifactorial nature of hepatocarcinogenesis.

The identification of these diverse risk factors presents a significant opportunity for targeted therapeutic interventions and preventative strategies. For instance, antiviral therapies and universal HBV vaccination programs have demonstrated substantial success in reducing virus‐associated HCC incidence. Similarly, lifestyle modifications—such as weight management, alcohol reduction, and smoking cessation—offer cost‐effective approaches to mitigating metabolic and environmental risks. Advances in molecular research, particularly in gut microbiota modulation and immune checkpoint inhibitors, hold promise for precision medicine, allowing for more effective, individualized treatments. Emerging targeted therapies, including tyrosine kinase inhibitors (e.g., sorafenib, lenvatinib), immune checkpoint inhibitors (e.g., nivolumab, pembrolizumab), and gut microbiota modulation, have shown potential in improving survival outcomes and reducing recurrence rates. The integration of these novel treatments with existing therapeutic strategies could further revolutionize HCC management.

Moreover, the integration of risk stratification tools and surveillance programs, including biomarker‐driven and imaging‐based monitoring, can enhance early detection, improving survival rates. Precision medicine approaches leveraging genetic and biomarker‐driven risk assessment are expected to play a critical role in tailoring treatments to individual patients, thereby improving therapeutic efficacy and minimizing adverse effects. Multidisciplinary collaborations among hepatologists, oncologists, geneticists, and epidemiologists are essential to translating these scientific advancements into clinical practice. Public health initiatives must also prioritize equitable healthcare access, especially in regions disproportionately affected by HCC, to bridge gaps in prevention and treatment.

As HCC incidence is projected to rise by 55% by 2040, proactive measures must be reinforced to curtail this trend. Future research should focus on unraveling the molecular mechanisms underlying emerging risk factors and developing novel, targeted therapies. Additionally, policymakers must implement stronger regulations on carcinogenic environmental exposures, improve access to preventive healthcare, and promote global vaccination initiatives. By leveraging these insights, the medical community can move closer to transforming HCC from a fatal malignancy into a preventable and more manageable disease, ultimately reducing the global burden of this devastating cancer.

## Author Contributions

Conceptualization: Muhammad Masroor Hussain, Hai‐jie Hu. Funding acquisition: Fu‐yu Li, Hai‐jie Hu. Writing – original draft: Muhammad Masroor Hussain. Writing – review and editing: Ju‐Mei Wang, Ao‐Qiang Zhai, Fu‐yu Li, Hai‐jie Hu.

## Ethics Statement

Since all analyses were based on publicly available summary statistics, no patients were involved in the design of the study, and no ethical approval from an institutional review board was required.

## Conflicts of Interest

The authors declare no conflicts of interest.

## Data Availability

Data sharing is not applicable to this article as no new data were created or analyzed in this study.
